# Supplementary material for: Ionic liquid enables highly efficient low temperature desalination by directional solvent extraction
Source: Nat Commun. 2021 Jan 19;12:437. doi: 10.1038/s41467-020-20706-y (PMC7815866; doi:10.1038/s41467-020-20706-y)
Supplement: Supplementary file 1 — Supplementary Information [file 41467_2020_20706_MOESM1_ESM.pdf]

Supplementary Information for

Ionic Liquid Enables Highly Efficient Low Temperature Desalination by Directional Solvent Extraction

Jiaji Guo,[a] Zachary D. Tucker,[b] Yu Wang,[a] Brandon L. Ashfeld,[b] Tengfei Luo[a,c]

[a] Department of Aerospace and Mechanical Engineering, University of Notre Dame, Notre Dame, IN 46556
[b] Department of Chemistry and Biochemistry, University of Notre Dame, Notre Dame, IN 46556
[c] Department of Chemical and Biomolecular Engineering, University of Notre Dame, Notre Dame, IN 46556

Emails: bashfeld@nd.edu; tluo@nd.edu

This PDF file includes:

Supplementary text
Figures S1 to S3
Tables S1 to S5
Supplementary Information References

Supplementary Information Text

1. SYNTHESIS AND CHARACTERIZATION OF ILS

General Methods: All organic solvents were distilled under and argon atmosphere, passed through a column of molecular sieves prior to use. Deionized water was used for all reactions unless otherwise stated. Reagents were used as received by commercial sources without further purification. All reactions were carried out in oven dried glassware under nitrogen or argon at room temperature unless otherwise specified. Compounds were characterized by ^{1}H nuclear magnetic resonance (NMR) spectra obtained at 400 or 500 MHz, ^{13}C NMR obtained at 100 or 125 MHz, and ^{19}F NMR obtained at 376 MHz. Chemical shifts are reported in parts per million (ppm, δ), and referenced from the solvent. Coupling constants are reported in Hertz (Hz). Spectral splitting patterns are designated as s, singlet; d, doublet; t, triplet; q, quartet; m, multiplet; comp, complex; app, apparent; and br, broad. Infrared (IR) spectra were obtained using a Thermo Electron Nicolet 380 FT-IR using a silicon (Si) crystal in an attenuated total reflectance (ATR) tower and reported as wavenumbers (cm-1). High- and Low-resolution electrospray ionization (ESI) measurements were made with a JEOL JMS-AX505HA mass spectrometer. Ionic Liquids: [emim][Tf_2N],[1] [P_{4444}][Tf_2N],[2] [P_{4444}][TsO],[3] [P_{4448}][BenzIm][4] and compounds tributyloctyl phosphonium bromide,[5] 1-butyl-3-methylimidazolium iodide,[6] and *N*-trifluoromethanesulfonylleucine methyl ester (I-Leu)[7] were synthesized by reported methods.

General Synthesis of Tetraalkylphosphonium bis(trifluoromethylsulfonyl)imide ionic liquids. To a 100 mL round bottom flask equipped with a magnetic stir bar was added tetraalkylphosphonium bromide (10 mmol, 1 equiv.), placed under an atmosphere of nitrogen, and diluted with H_2O (40 mL). To the solution of tetralkylphosphonium bromide was added $LiTf_2N$ (10 mmol, 1 equiv.) as a 1M solution in H_2O. The mixture was stirred at rt for 15 hours then extracted with CH_2Cl_2 (3 x 20 mL). The combined organic fractions were washed with H_2O (3 x 10 mL), dried, ($MgSO_4$), filtered, and concentrated under reduced pressure. The ionic liquid was then dried further for an additional 12 hours in a vacuum oven set to ~80 °C to provide the desired compound.

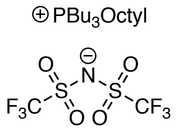

[P_{4448}][Tf_2N]

Tributyloctylphosphonium bis(trifluoromethanesulfonyl)imide [P_{4448}][Tf_2N]: The synthesis of [P_{4448}][Tf_2N] was conducted on a 10 mmol scale using tributyloctyl phosphonium bromide to provide 5.60 g (92% yield) of the title compound as a clear colorless oil. ^{1}H NMR (400 MHz, $CDCl_3$) δ 2.20-2.02 (m, 8H), 1.62-1.38 (m, 16 H), 1.36-1.21 (m, 8 H), 0.97 (t, J = 8.0 Hz, 9 H), 0.88 (t, *J* = 8.0 Hz, 3 H). ^{13}C NMR (100 MHz, $CDCl_3$) δ 119.9 (q, *J* = 321.7 Hz), 31.9, 30.9, 30.3, 30.1, 29.7, 29.4, 29.3, 28.8, 22.7, 22.3, 21.5, 18.9, 18.4, 13.9. ^{19}F NMR (376 MHz, $CDCl_3$) δ -78.85.

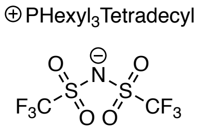

[P_{66614}][Tf_2N]

Trihexyltetradecylphosphonium bis(trifluoromethanesulfonyl)imide [P_{66614}][Tf_2N]: The synthesis of [P_{66614}][Tf_2N] was conducted on a 10 mmol scale using trihexyltetradecyl phosphonium bromide to provide 6.49 g (85% yield) of the title compound as a clear colorless oil. ^{1}H NMR (400 MHz, $CDCl_3$) δ 2.17-2.01 (m, 8 H), 1.56-1.38 (m, 16 H), 1.37-1.18 (m, 32H), 0.88 (t, *J* = 7.1 Hz, 12 H). ^{13}C NMR (100 MHz, $CDCl_3$) δ 119.9 (q, *J* = 321.6 Hz), 32.0, 30.9, 30.6, 30.5, 30.3, 30.1, 29.7, 29.7, 29.6, 29.5, 29.4, 29.3, 28.8, 22.7, 22.3, 21.5, 18.9, 18.4, 14.1, 13.9. ^{19}F NMR (376 MHz, $CDCl_3$) δ -78.82.

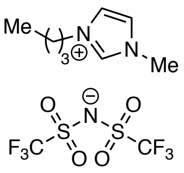

[bmim][Tf$_2$N]

1-Butyl-3-methylimidazolium bis(trifluoromethanesulfonyl)imide [bmim][Tf$_2$N]: To a 10 mL round bottom flask equipped with a magnetic stir bar was added 1-butyl-3-methylimidazolium iodide (4.38 g, 20 mmol, 1 equiv.), placed under an atmosphere of N_2 then diluted with H_2O (5 mL). To the resulting solution was added $LiTf_2N$ (6.03 g, 21 mmol, 1.05 equiv.) as a 6M solution in H_2O. The mixture was stirred at rt for 15 hours then extracted with CH_2Cl_2 (3 x 15 mL). The combined organic fractions were washed with H_2O (3 x 10 mL), dried ($MgSO_4$), filtered, and concentrated under reduced pressure. The ionic liquid was then dried further for an additional 12 hours in a vacuum oven set to ~80 °C to provide 8.14 g (97% yield) of the title compound as a faintly yellow oil. ^{1}H NMR (400 MHz, $CDCl_3$) δ 8.73 (s, 1H), 7.31 (s, 2 H), 4.17 (t, *J* = 7.5 Hz, 2 H), 3.93 (s, 3 H), 1.85 (p, *J* = 7.5 Hz, 2 H), 1.36 (h, *J* = 7.4 Hz, 3 H), 0.96 (t, *J* = 7.4 Hz, 3 H). ^{13}C NMR (100 MHz, $CDCl_3$) δ 136.07, 123.67, 122.24, 119.79 (q, *J* = 320.0 Hz), 49.94, 36.31, 31.92, 19.32, 13.20. ^{19}F NMR (376 MHz, $CDCl_3$) δ -79.11.

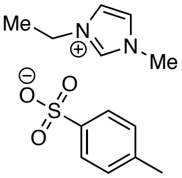

[emim][TsO]

1-Ethyl-3-methylimidazolium tosylate [emim][TsO]: To a 50 mL round bottom flask equipped with a magnetic stir bar was added 1-ethyl-3-methylimidazolium chloride (1.47 g, 10 mmol, 1 equiv.), placed under an atmosphere of N_2 then diluted with MeCN (10 mL) and Et_2O (5 mL). To the resulting solution was added AgTsO (2.79 g, 10 mmol, 1 equiv.). The mixture was stirred at rt for 1 hour then filtered through a bed of Celite to remove AgCl. The filter cake was washed with MeCN/Et_2O (1:1) (3 x 10 mL), then the filtrate was concentrated under reduced pressure. The crude oil was reconstituted in CH_2Cl_2 (50 mL) then washed with H_2O (3 x 10 mL), dried ($MgSO_4$), filtered, and concentrated under reduced pressure. The ionic liquid was then dried further for an additional 12 hours in a vacuum oven set to ~80 °C to provide 2.74 g (97% yield) of the title compound as a yellow oil. ^{1}H NMR (400 MHz, Acetone-*d*$_6$) δ 9.57 (s, 1H), 7.78 (d, *J* = 32.5 Hz, 2 H), 7.67 (d, *J* = 8.2 Hz, 2 H), 7.13 (d, *J* = 7.4 Hz, 2 H), 4.31 (q, *J* = 7.3 Hz, 2 H), 3.97 (s, 3 H), 2.30 (s, 3 H), 1.44 (t, *J* = 7.3 Hz, 3 H). ^{13}C NMR (100 MHz, Acetone-*d*$_6$) δ 147.2, 138.9, 138.3, 129.0, 126.7, 124.5, 122.9, 45.4, 36.4, 21.2, 15.8.

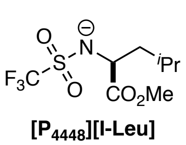

[P$_{4448}$][l-Leu]

Tributyloctylphosphonium trifluoromethanesulfonylleucine methyl ester salt [P$_{4448}$][l-Leu]: To a 20 mL round bottom flask equipped with a magnetic stir bar was added tributyl(octyl)phosphonium bromide (3.57 g, 9 mmol, 1 equiv.), placed under an atmosphere of N_2, then diluted with MeOH (9 ml). To the resulting solution was added 2M methanolic KOH (9.1 mmol, 5.1mL), precipitation of KBr was observed. The suspension was stirred at room temperature for 12 hours, then filtered through a bed of Celite to remove KBr. The filter cake washed with MeOH (3 x 5 mL) and to the filtrate was added *N*-trifluoromethanesulfonylleucine methyl ester (2.77g, 9 mmol, 1 equiv.) and stirred at rt for 12 hours. The resulting solution was concentrated under reduced pressure and reconstituted in acetone (10 mL), filtered through a bed of Celite, then the resulted

filtrate was concentrated under reduced pressure. The ionic liquid was then dried further for an additional 48 hours in a vacuum oven set to ~80 °C to provide 3.90 g (75 % yield) of the title compound as a yellow oil. ^{1}H NMR (400 MHz, $CDCl_3$) δ 4.07 (dd, *J* = 8.0, 5.8 Hz, 1 H), 3.66 (s, 3 H), 2.30 (dp, *J* = 19.0, 6.5, 5.8 Hz, 7 H), 1.80 (dp, *J* = 19.0, 6.5 Hz, 1H), 1.67 -1.39 (m, 18 H), 1.39-1.17 (m, 8 H), 0.97 (t, *J* = 8.0 Hz, 9 H), 0.95-0.82 (m, 10H). ^{13}C NMR (100 MHz, $CDCl_3$) δ 175.4, 121.7 (q, *J* = 326.0 Hz), 57.0, 51.5, 44.2, 30.8, 30.7, 29.0, 24.4, 24.0, 23.8, 23.7, 23.0, 22.6, 21.8, 18.9, 18.4, 14.1, 13.5. ^{19}F NMR (376 MHz, $CDCl_3$) δ -77.21. IR (neat) 2931, 1740, 1466, 1368, 1266, 1193, 1087, 912 cm^{-1}; HRMS (ESI) m/z: measured 315.318086 $C_{20}H_{44}P^{+}$ (M) requires 315.317514, HRMS (ESI) m/z: measured 276.051640 $C_8H_{13}F_3NO_4S$ (M) requires 276.052287.

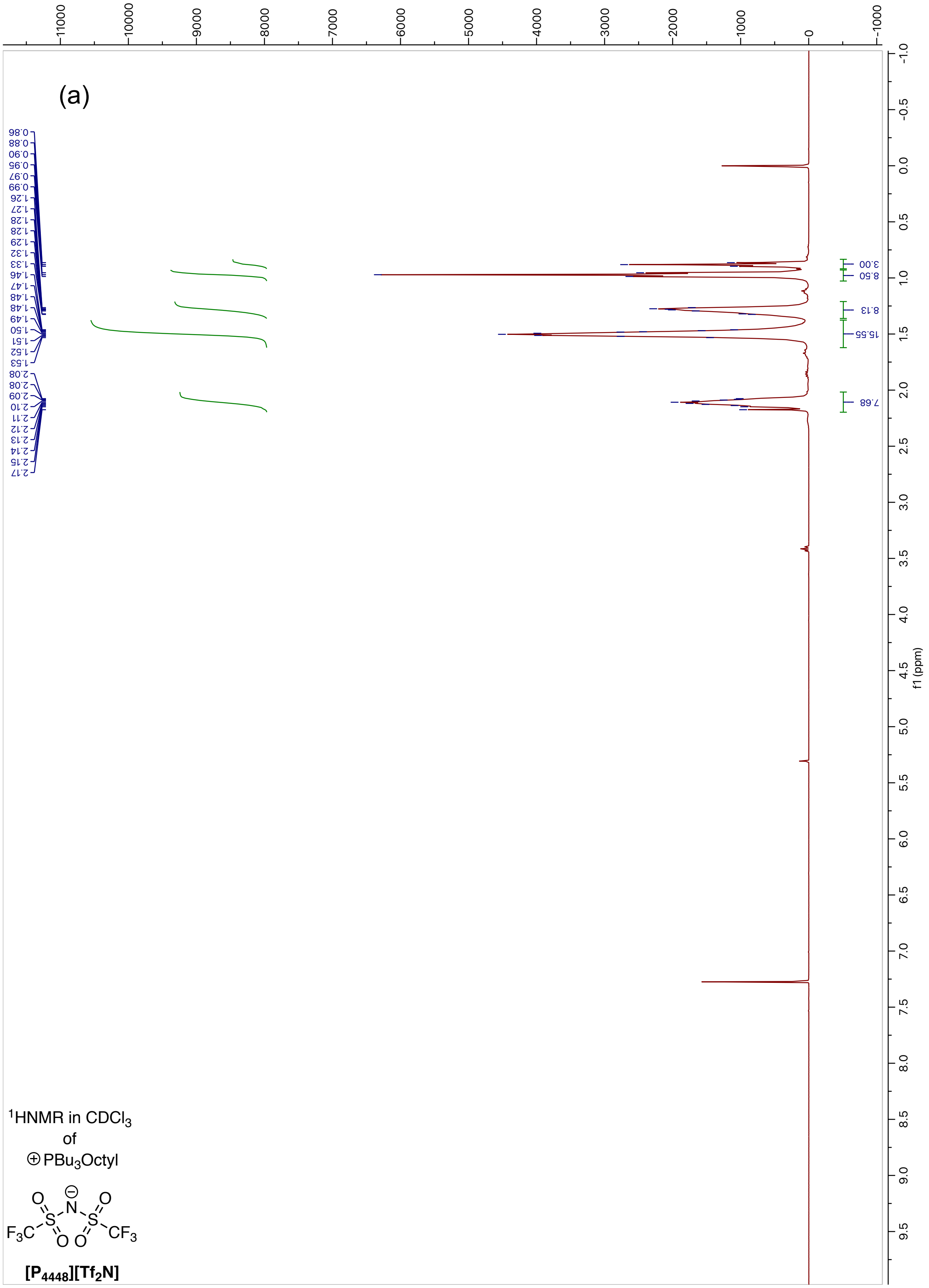

(a)
¹HNMR in CDCl3
of
⊕PBu3Octyl
[P4448][Tf2N]
f1 (ppm)
3.00
8.50
8.13
15.55
7.68

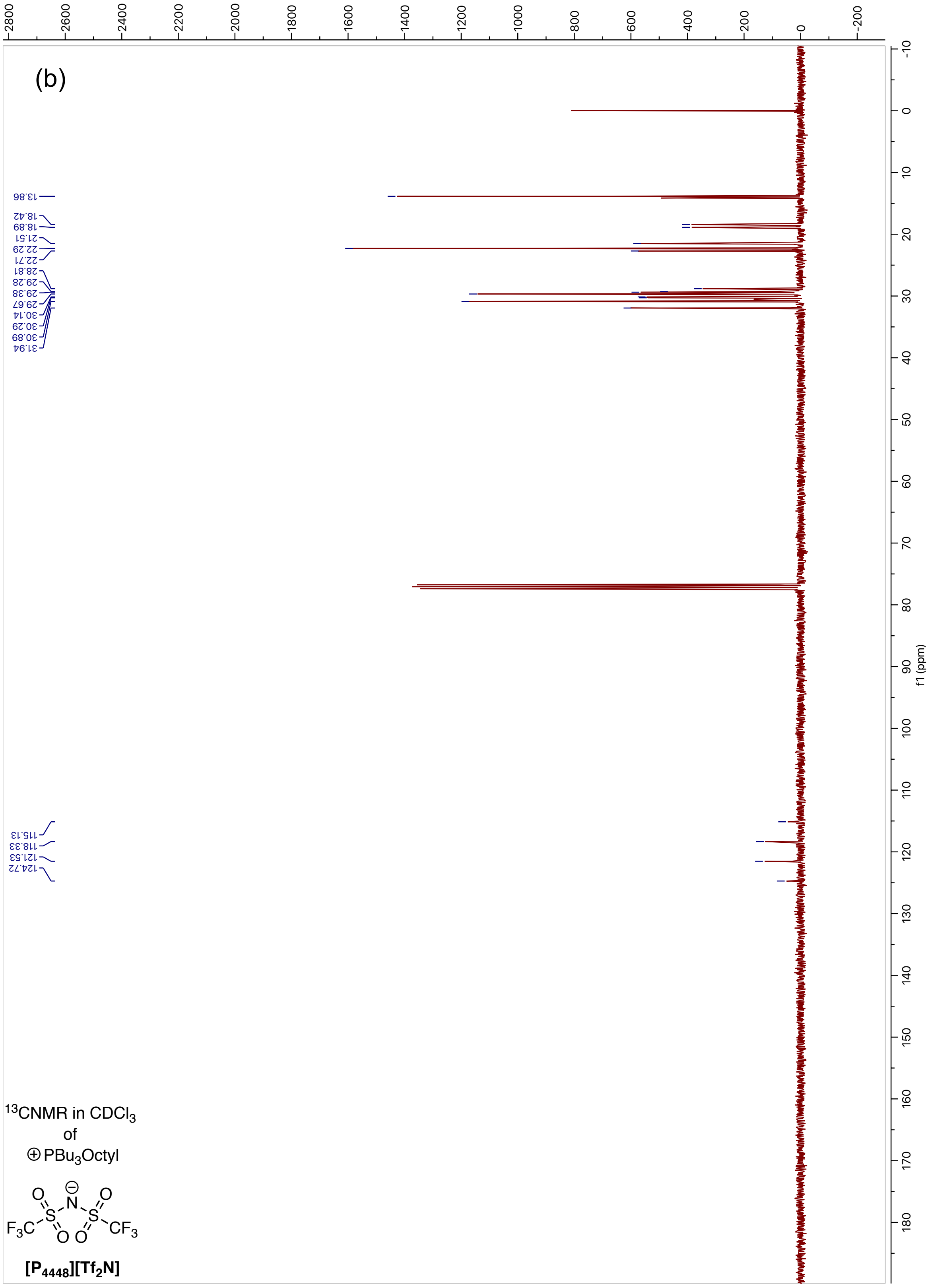
(b)
13.86
18.42
18.89
21.51
22.29
22.71
28.81
29.28
29.38
29.67
30.14
30.29
30.89
31.94
115.13
118.33
121.53
124.72
f1 (ppm)
13CNMR in CDCl3
of
⊕PBu3Octyl
[P4448][Tf2N]

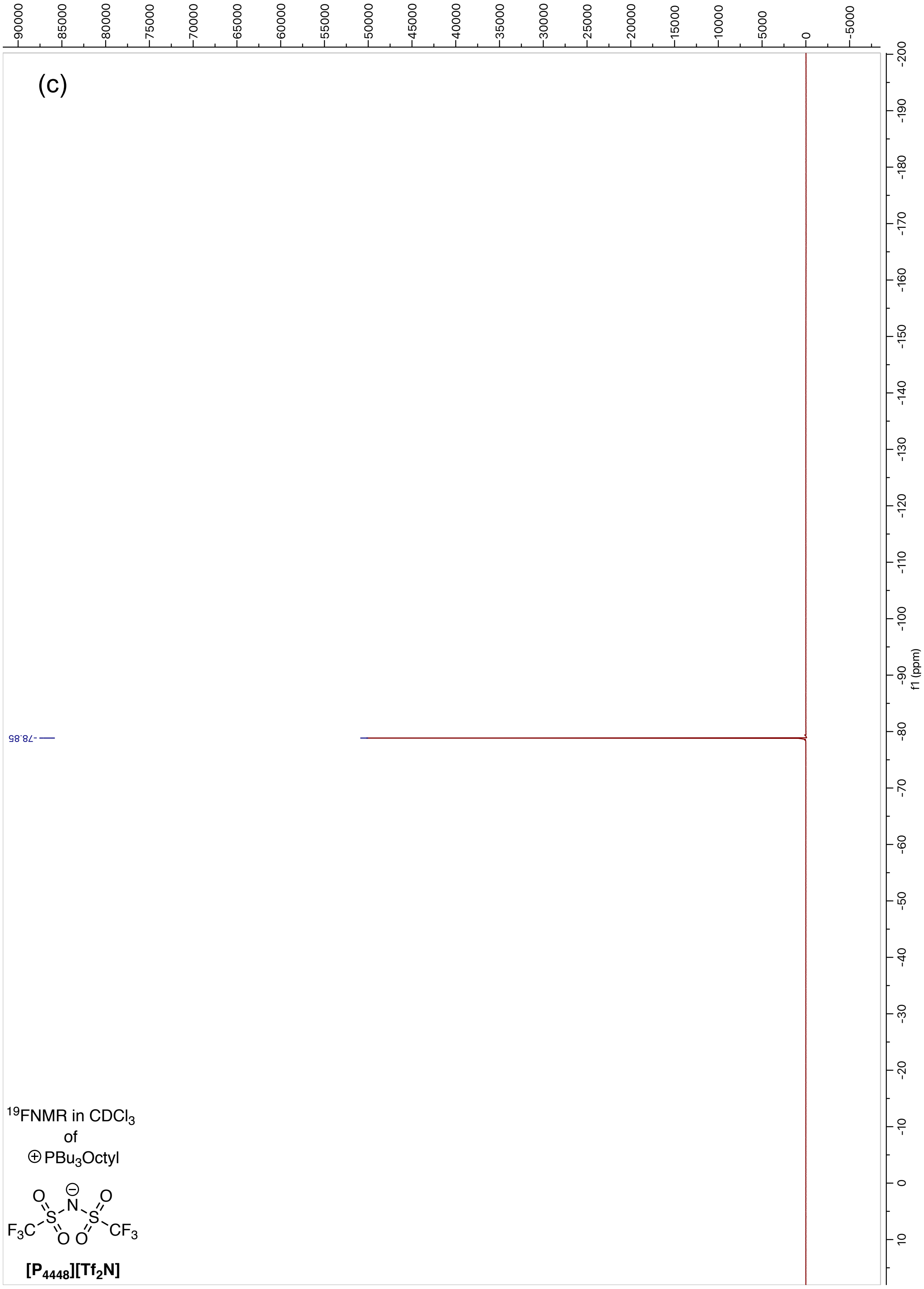
(c)
-78.85
f1 (ppm)
^{19}FNMR in $CDCl_3$
of
⊕PBu$_3$Octyl
F_3C CF_3
$[P_{4448}][Tf_2N]$

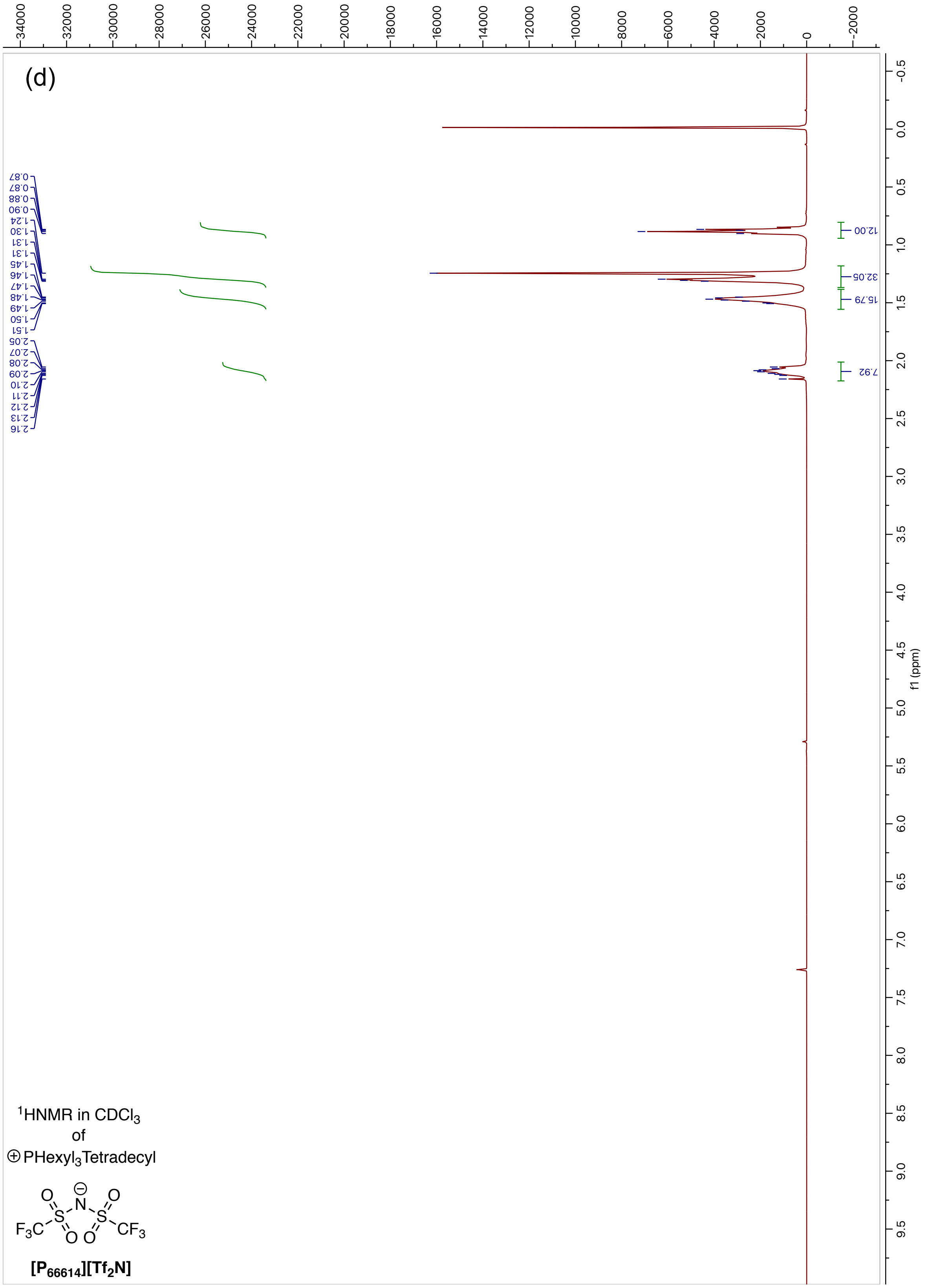

(d)
¹HNMR in CDCl3
of
⊕PHexyl3Tetradecyl
[P66614][Tf2N]
12.00
32.05
15.79
7.92
f1 (ppm)

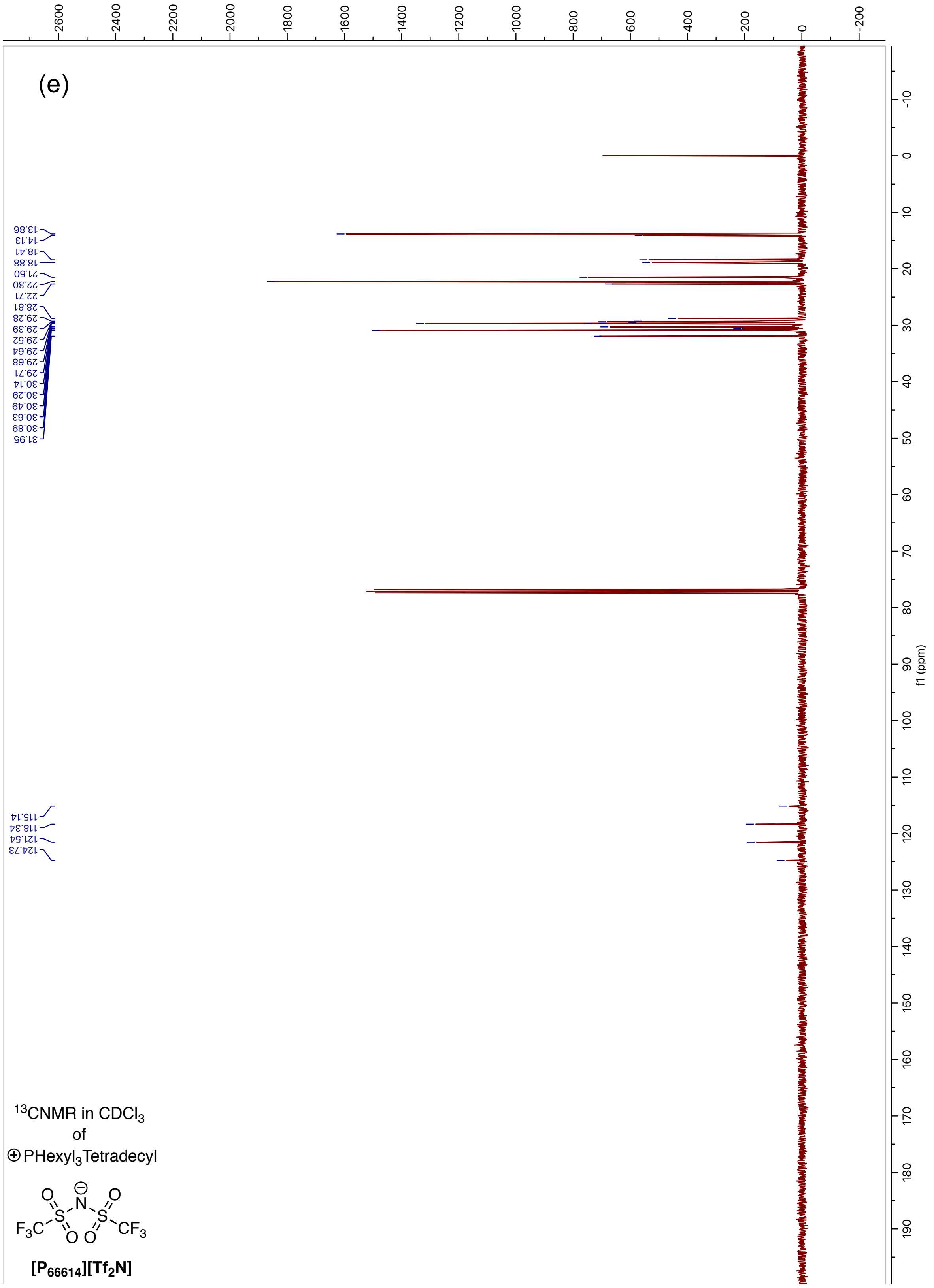
(e)
13CNMR in CDCl3
of
⊕PHexyl3Tetradecyl
[P66614][Tf2N]
f1 (ppm)

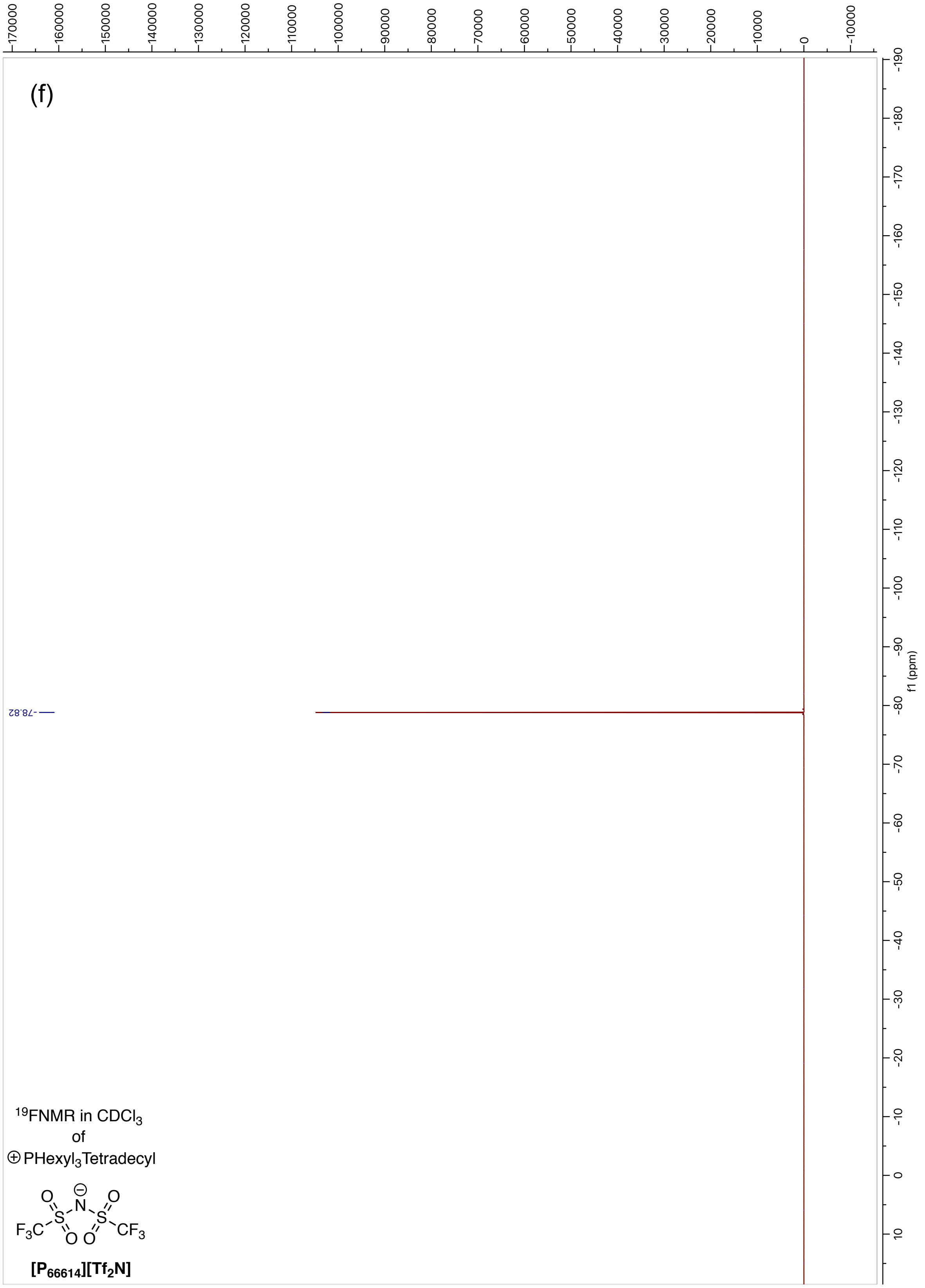
(f)
—-78.82
f1 (ppm)
19FNMR in CDCl3
of
⊕PHexyl3Tetradecyl
[P66614][Tf2N]

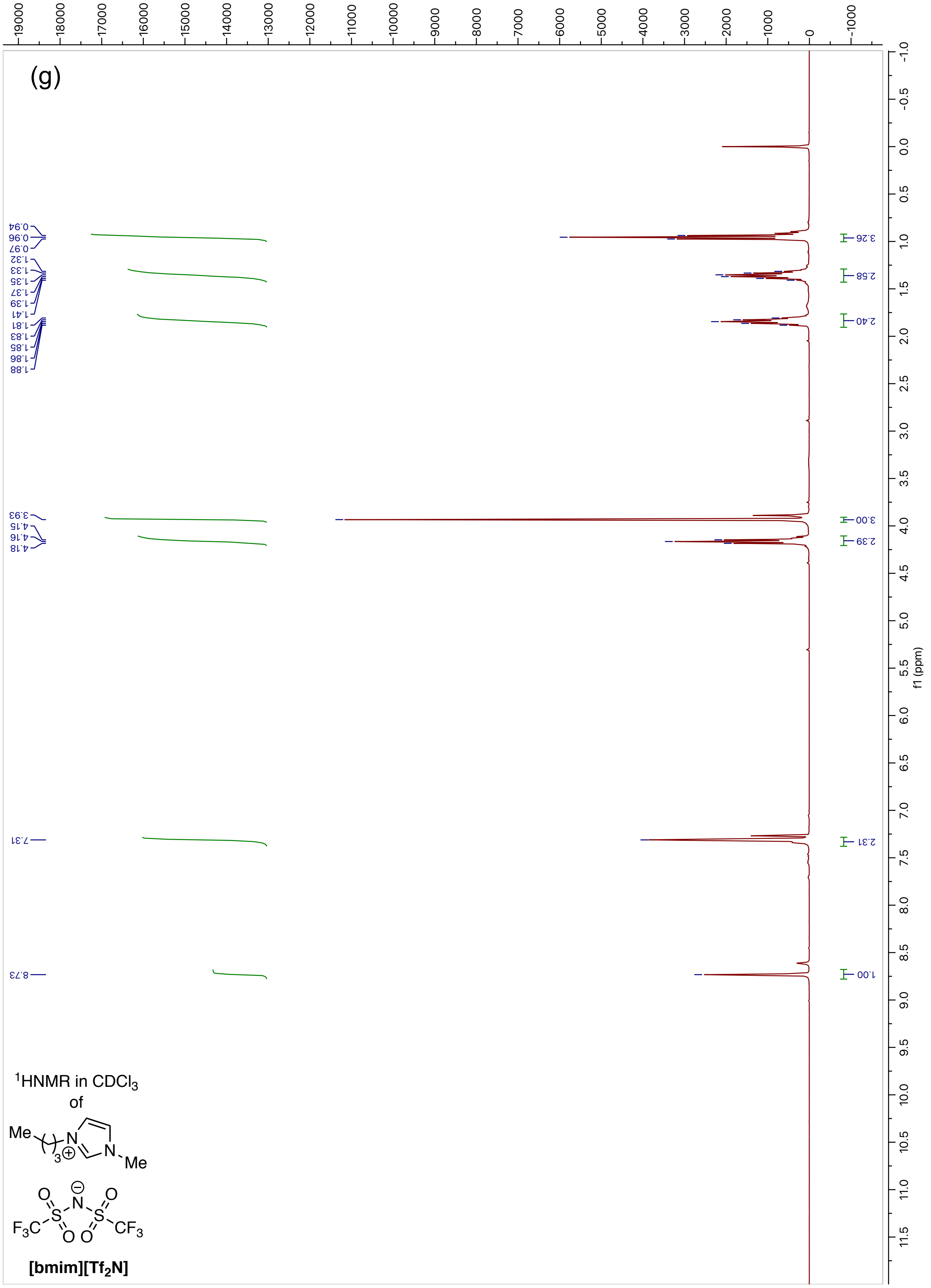
(g)
1HNMR in CDCl3
of
[bmim][Tf2N]
f1 (ppm)

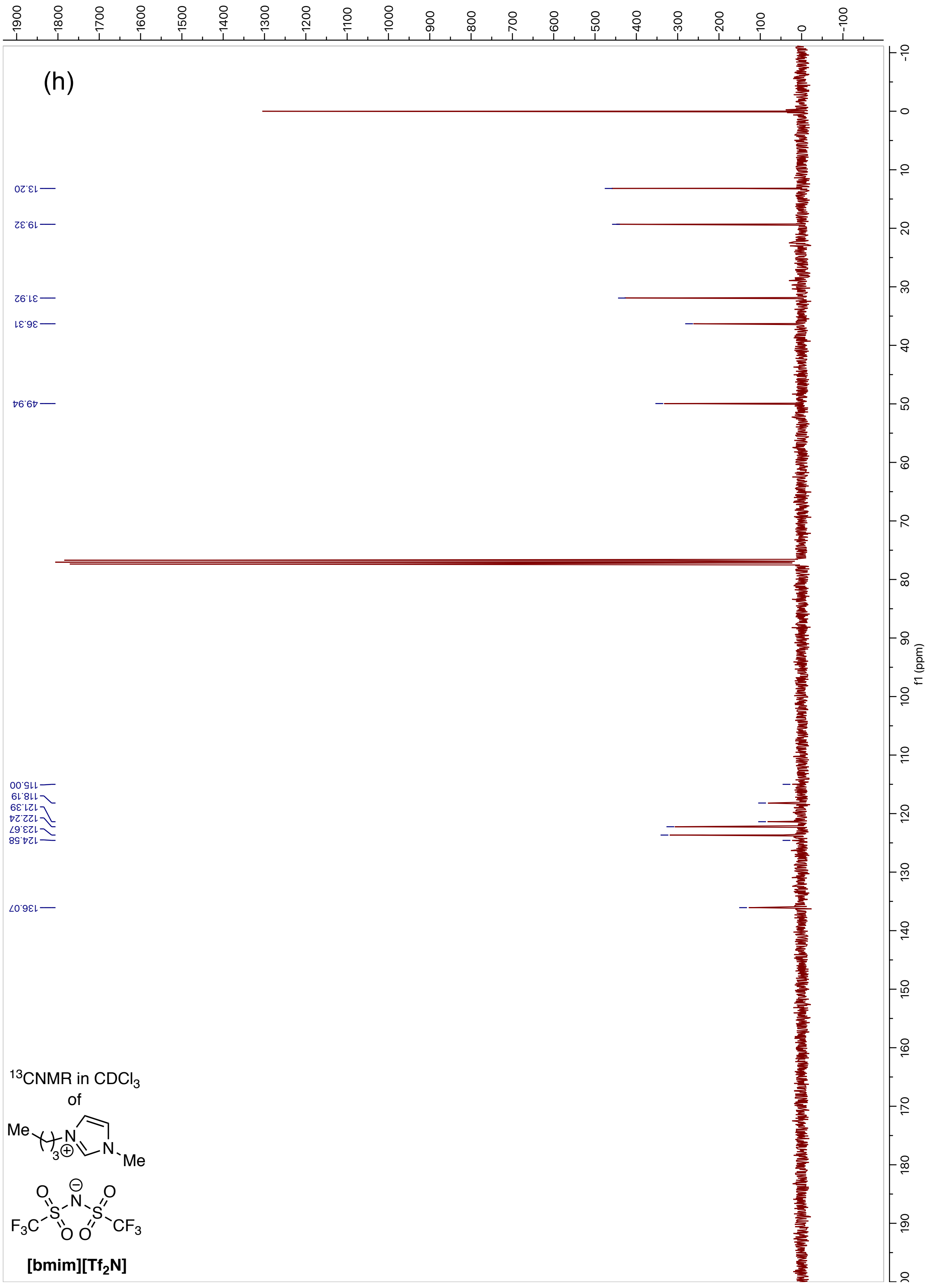
(h)
13.20
19.32
31.92
36.31
49.94
115.00
118.19
121.39
122.24
123.67
124.58
136.07
f1 (ppm)
13CNMR in CDCl3
of
[bmim][Tf2N]

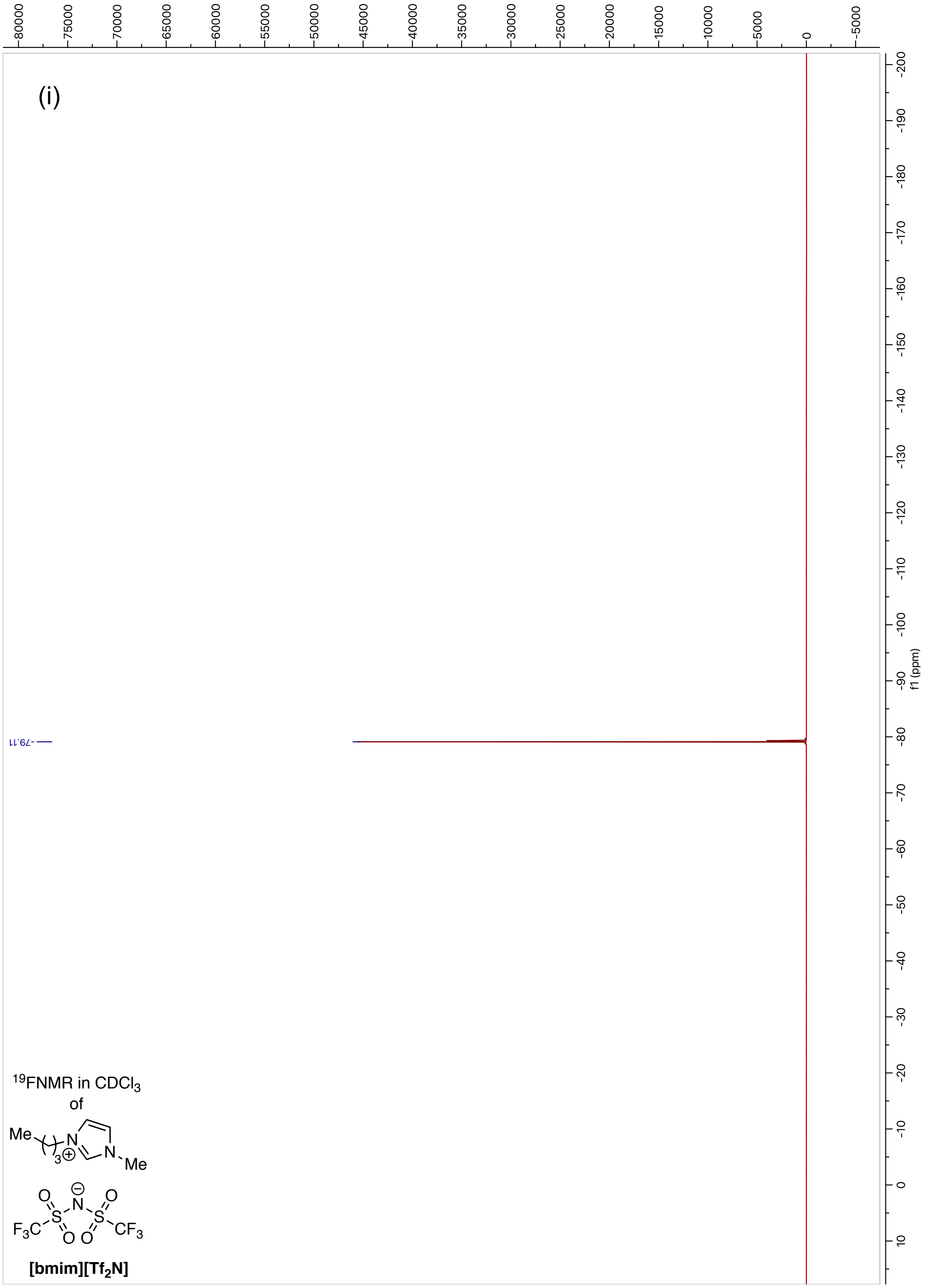
(i)
-79.11
f1 (ppm)
^{19}FNMR in $CDCl_3$
of
[bmim][Tf$_2$N]

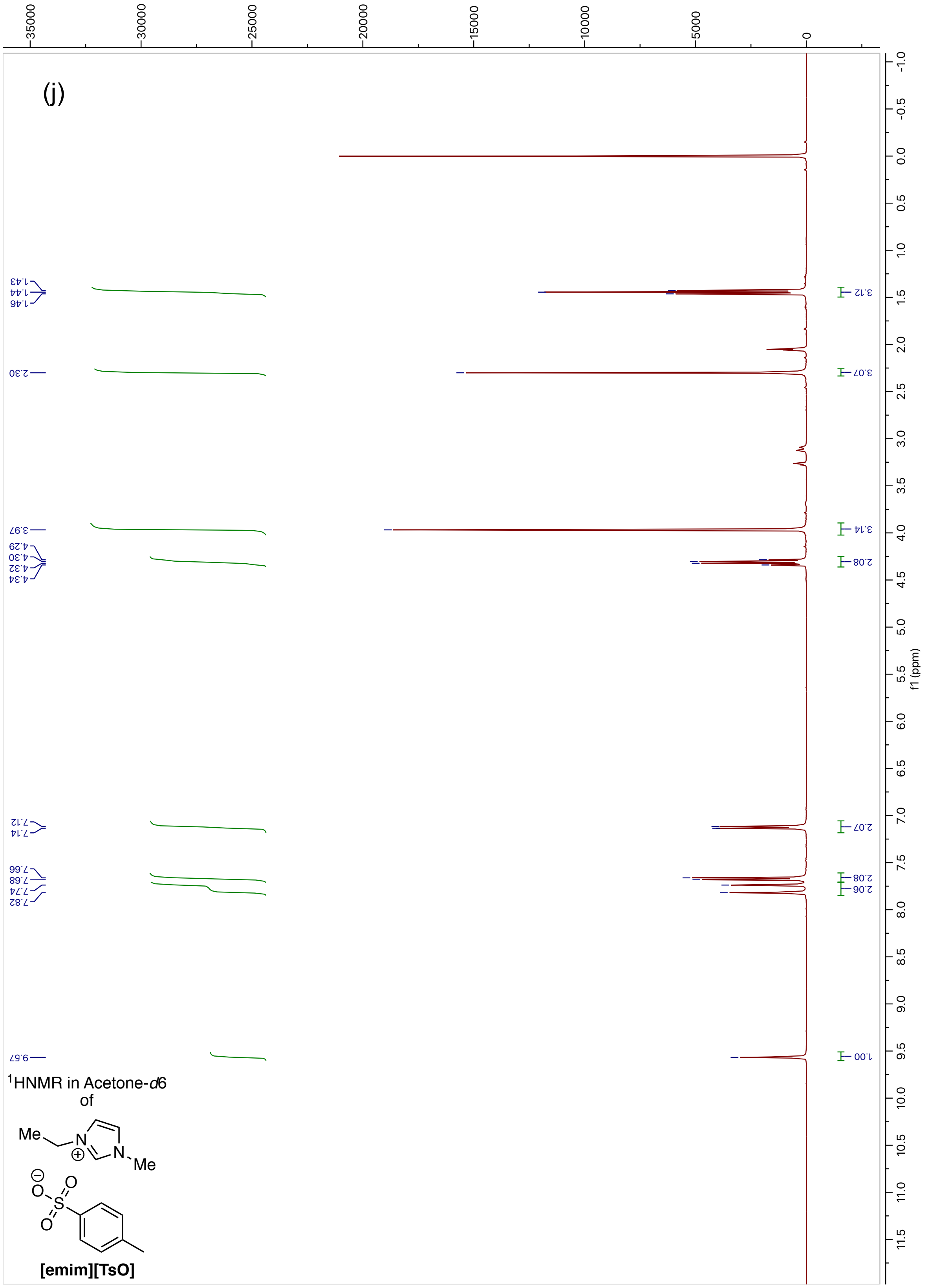
(j)
1HNMR in Acetone-d6
of
Me
N
N
Me
O
O
S
O
[emim][TsO]
1.43
1.44
1.46
2.30
3.97
4.29
4.30
4.32
4.34
7.12
7.14
7.66
7.68
7.74
7.82
9.57
3.12
3.07
3.14
2.08
2.07
2.08
2.06
1.00
f1 (ppm)

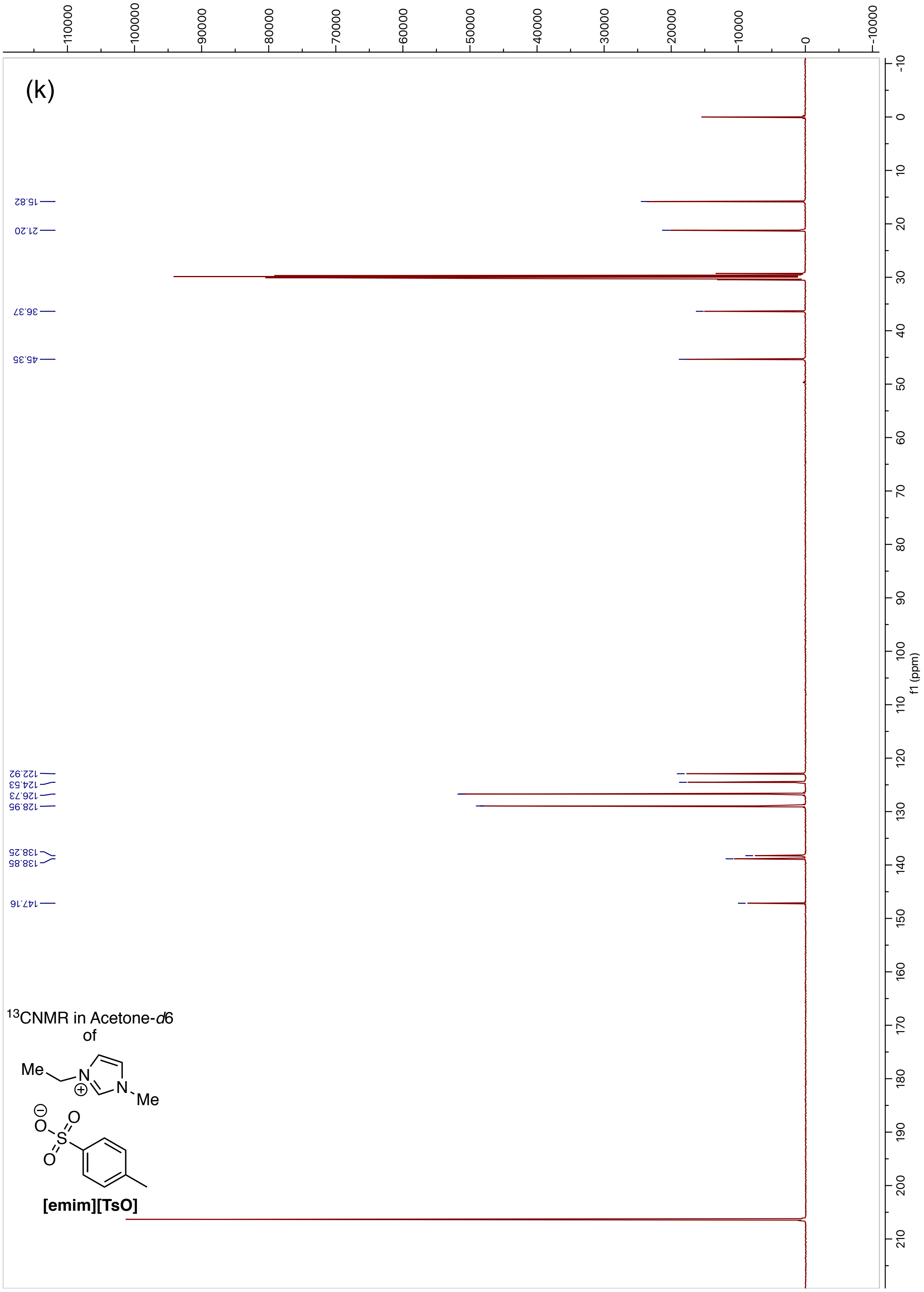
(k)
15.82
21.20
36.37
45.35
122.92
124.53
126.73
128.95
138.25
138.85
147.16
f1 (ppm)
13CNMR in Acetone-d6
of
[emim][TsO]

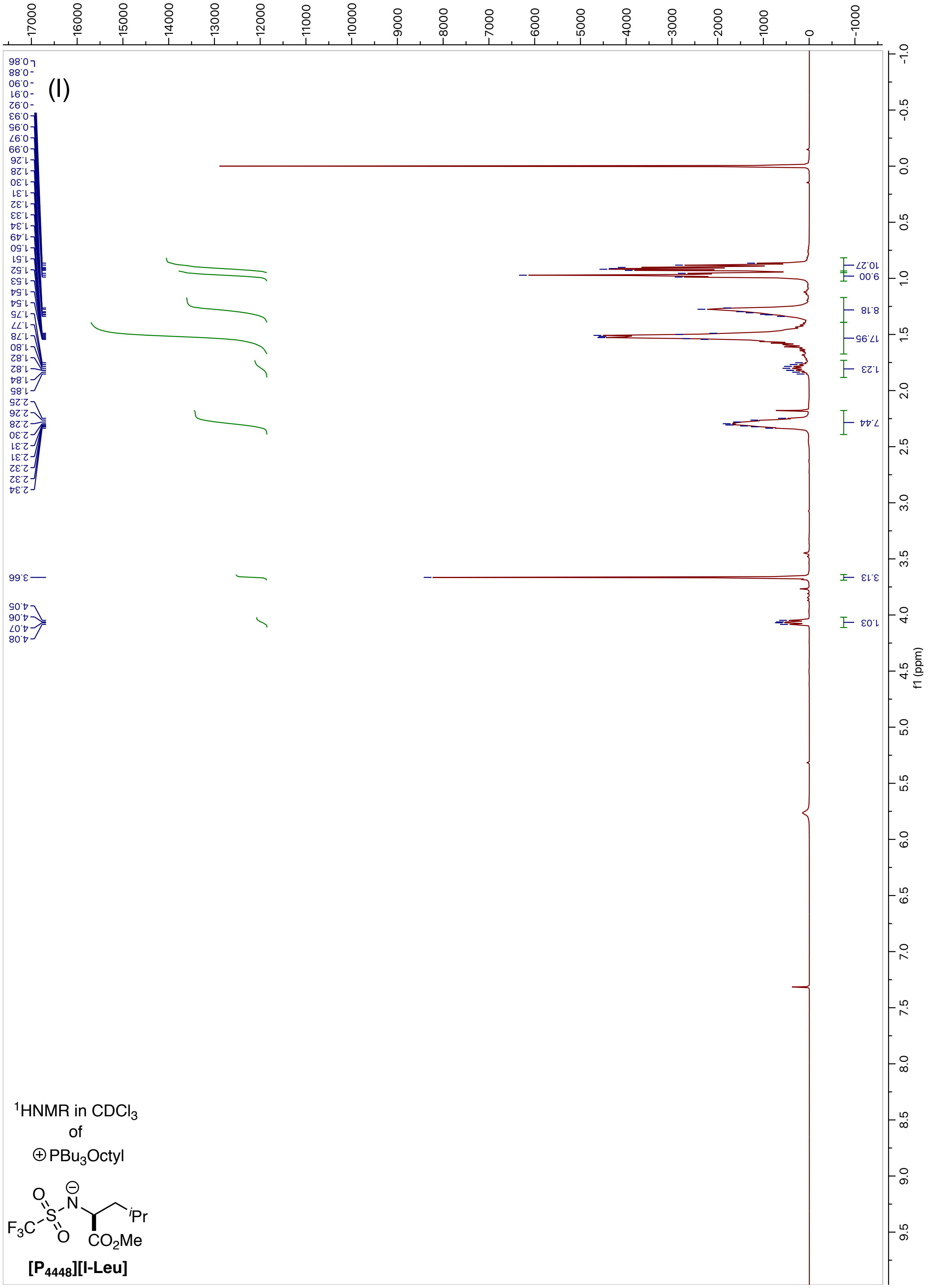
(I)
1HNMR in CDCl3
of
⊕PBu3Octyl
[P4448][I-Leu]
f1 (ppm)

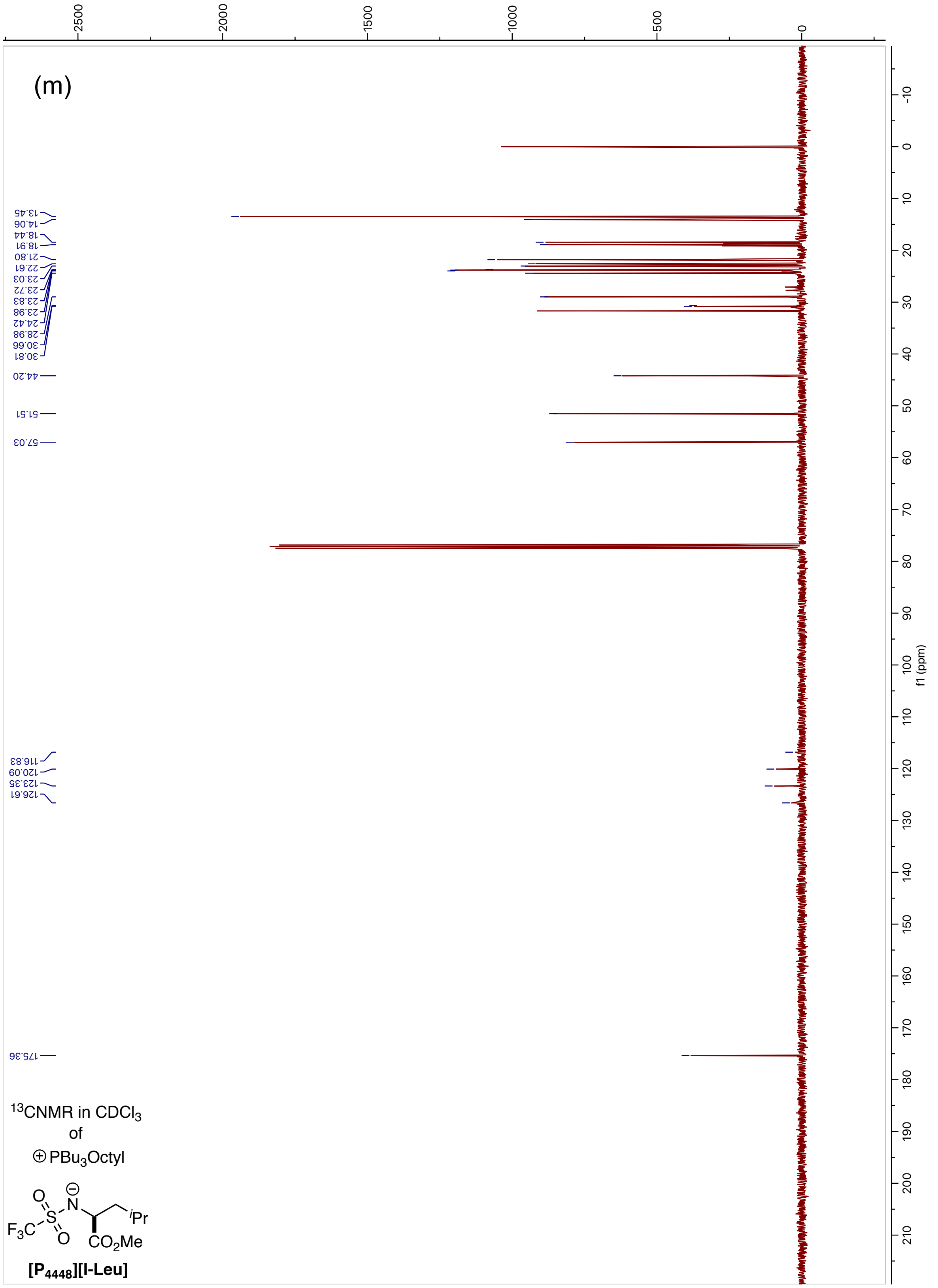
(m)
13.45
14.06
18.44
18.91
21.80
22.61
23.03
23.72
23.83
23.98
24.42
28.98
30.66
30.81
44.20
51.51
57.03
116.83
120.09
123.35
126.61
175.36
f1 (ppm)
13CNMR in CDCl3
of
⊕PBu3Octyl
[P4448][I-Leu]

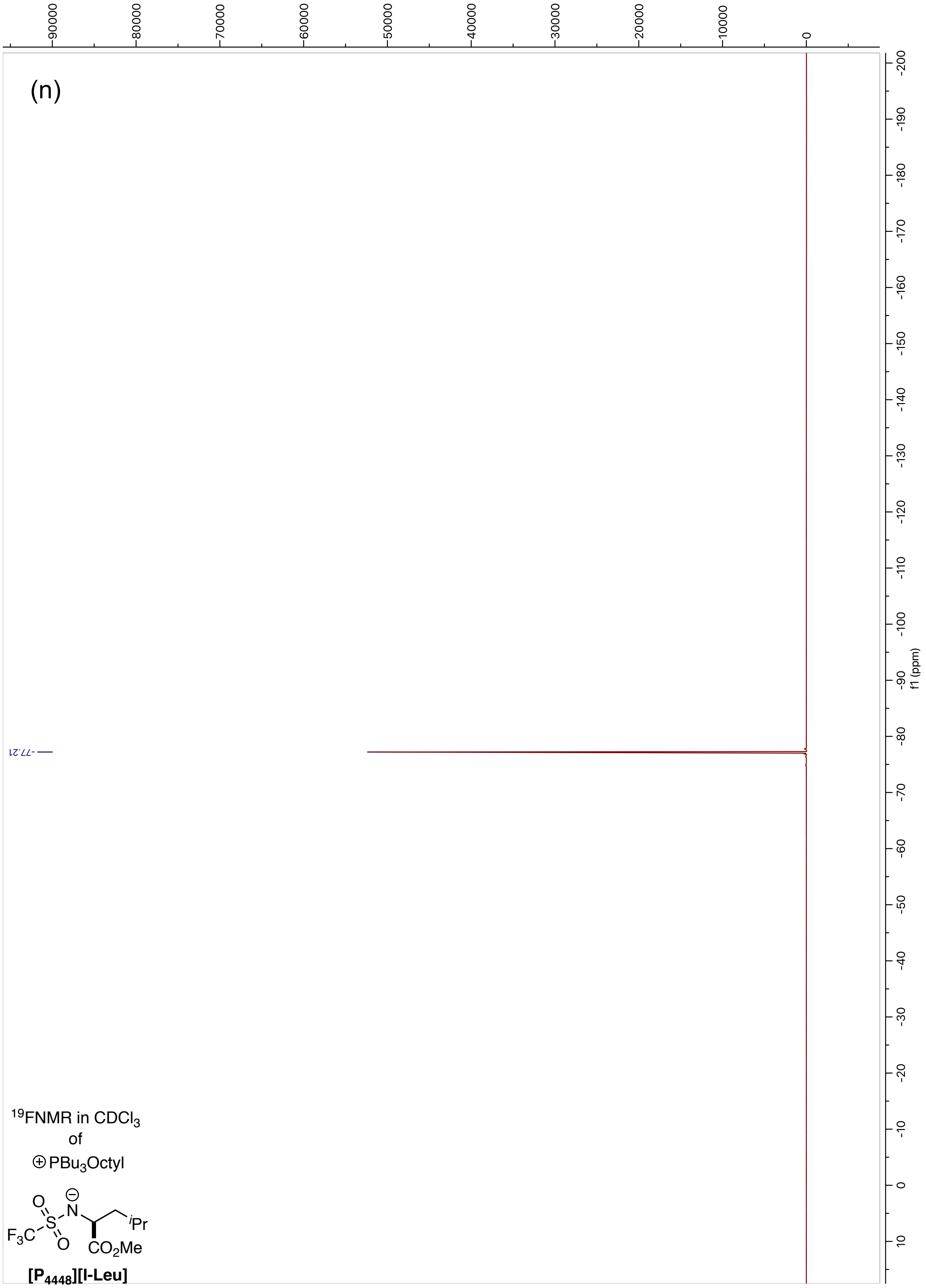
(n)
-77.21
f1 (ppm)
19FNMR in CDCl3
of
PBu3Octyl
[P4448][l-Leu]

Figure S1. (a) to (m) NMR spectroscopy of synthesized substances.

2. DSE EXPERIMENTAL SCREENING OF CANDIDATE ILS

The experimental procedure is briefly depicted in Fig. S2. First, 2 mL of an IL and 2 mL of saline water with 3.7-5.0 wt% NaCl are mixed thoroughly at an elevated temperature (*e.g.*, 45, 60 and 75 °C). The mixture is maintained at that temperature for ~10 minutes for phase separation, expecting the brine to separate from the IL. The IL phase, which is supposed to have pure water dissolved in it, is collected and cooled down to room temperature, expecting the fresh water to precipitate out due to lowered solubility in the IL. The separation of these two phases is facilitated by a centrifuge at 3000 rpm. The amount of recovered water is used to calculate the yield, and the water is also characterized for contents of NaCl ions and residual ILs to verify directional solubilities. The NaCl ion concentration is measured using Perkin Elmer Optima 8000 ICP-OES and the IL content is measured using Waters TQD triple mass spectrometer coupled to an Acquity ultrahigh pressure liquid chromatography system.

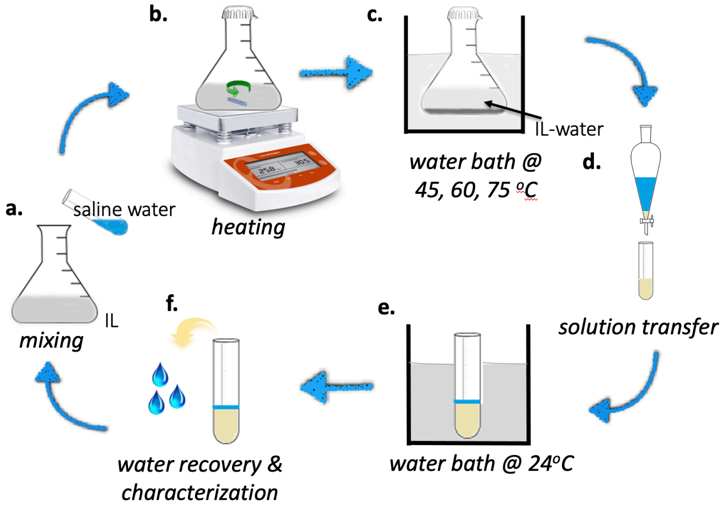

Figure S2. Experimental DSE procedure of water desalination using ILs.

3. CONCENTRATION MEASUREMENT OF NACL, [EMIM][TF$_2$N], AND [BMIM][TF$_2$N]

All Na^+ concentration measurements are performed using Perkin Elmer Optima 8000 ICP-OES. Since all water samples in the experiment are deionized water, it is rational to assume that the molar concentration of Na^+ is equal to that of NaCl. The Perkin Elmer Optima 8000 has a detection limit of ~10 ppb, which is accurate enough to detect the concentration of NaCl in the recovered water and [emim][Tf$_2$N]. A set of calibration curves of conductivity versus Na^+ ion concentration are established prior to the measurements. The detection of the concentration of [emim][Tf$_2$N] and [bmim][Tf$_2$N] in water is performed on the Waters TQD triple quadrupole mass spectrometer coupled to an Acquity ultrahigh pressure liquid chromatography system. Samples with different IL/water ratios are mixed in beaker with stirring speed of 300 rpm for 10 hours, and then a 3-hour sitting period is allowed to guarantee thermodynamic equilibrium of dissolution. Because the ILs tested have larger density than water, the solution in the upper part of the beaker is extracted as the sample. Concentration of $[Tf_2N]^-$ is tested in mass spectrometer to calculate the IL solubility in water. The intensity of a certain ion acquired by the Mass Spectrometer proportionally reflects the concentration of this kind of ion in the water solution.

4. LIST OF IONIC LIQUIDS TESTED IN EXPERIMENT

All the ILs studied in this work are listed in Table S1. The information of the ILs tested to be incompatible with DSE is listed in Table S2 and the viscosity of the ILs is listed in Table S3. The viscosity is measured with Discovery Hybrid Rheometer 2.

Table S1. General information of ILs used

Full Name	Abbreviation	structure	Purity[1]	CAS number
1-ethyl-3-methylimidazolium bis(trifluoromethylsulfonyl)imide	[emim][Tf$_2$N]	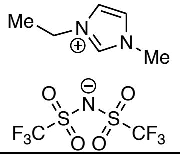	>99%	174899-82-2
1-butyl-3-methylimidazolium bis(trifluoromethylsulfonyl)imide	[bmim][Tf$_2$N]	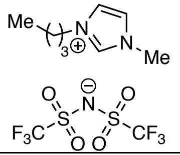	>99%	174899-83-3
1-Ethyl-3-methylimidazolium tosylate	[emim][TsO]	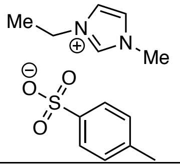	>99%	328090-25-1
Tetrabutylphosphonium tosylate	[P$_{4444}$][TsO]	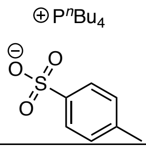	>99%	116237-97-9
Tetrabutylphosphonium bis(trifluoromethylsulfonyl)imide	[P$_{4444}$][Tf$_2$N]	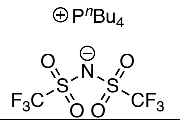	>99%	210230-40-3
Trihexyltetradecylphosphonium bis(trifluoromethanesulfonyl)imide	[P$_{66614}$][Tf$_2$N]	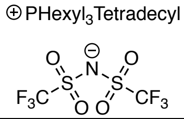	>99%	460092-03-9
Tributyloctylphosphonium bis(trifluoromethanesulfonyl)imide	[P$_{4448}$][Tf$_2$N]	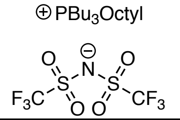	>99%	
Tributyloctylphosphonium benzo[*d*]imidazol-1-ide	[P$_{4448}$][BzIm]	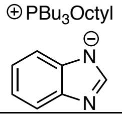	>99%	
Tributyloctylphosphonium trifluoromethanesulfonylleucine	[P$_{4448}$][I-Leu]	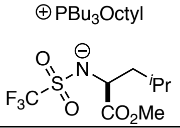	>99%	

[1]Determined via ^{1}HNMR

Table S2. TSIL DSE Performance

Solvent	Performance
[emim][TsO]	Precipitate formation
[P_{4444}][TsO]	Precipitate formation
[P_{4444}][Tf_2N]	High melting temperature (~65°C)
[P_{66614}][Tf_2N]	High viscosity, low water yield (<0.012%/°C)
[P_{4448}][Tf_2N]	High viscosity, low water yield (<0.005%/°C)
[P_{4448}][BzIm]	High viscosity
[P_{4448}] l-Leu	High viscosity

Table S3. ILs' Viscosity at 293K

Solvent	Viscosity in Pa·s
[emim][Tf_2N]	0.033 ± 0.014
[bmim][Tf_2N]	0.056 ± 0.010
[P_{66614}][Tf_2N]	0.081 ± 0.059
[P_{4448}][Tf_2N]	0.433 ± 0.099
[P_{4448}][BzIm]	0.185 ± 0.092
[P_{4448}] l-Leu	1.017 ± 0.078

It is shown that all [emim][Tf_2N], [bmim][Tf_2N] and [P_{66614}][Tf_2N] viscosities are in the similar range as those in the literature.[8-10]

5. MOLECULAR DYNAMICS SIMULATION DETAILS

We choose the GROMACS (Groningen Machine for Chemical Simulations) package to perform MD simulation.[11] The all-atom optimized potential for liquid simulation[12] (OPLS-aa) force filed is used for [emim][Tf_2N]. We list all parameters and status settings used for the simulations in Table S4. The TIP3P model and TIP4P model are separately used for water molecules to ensure that the calculated free energy tendencies are independent of force field (Table S5).[13, 14] In the simulations, we use a cutoff of 1.0 nm for van der Waals (vdW) and short-range electrostatic interaction. For the long range electrostatic interactions, the Fast Particle-Mesh Ewald (PME)[15] method with a 0.12 nm spacing for the fast - Fourier transformation (FFT) grid and a 6th order interpolation scheme are used. All bonds are constrained by the Parallel Linear Constraint Solver (P-LINCS),[16] and we use a time step of 2 fs for all simulations. We build Cubic simulation boxes with sides of ~20 nm to guarantee that there is no interaction between the ion and its images in the periodic cells. The simulation of a dilute limit is empowered by this.

Table S4. Forcefield parameters in the simulation

Parameter	Value/Status
Coulomb type	PME
Coulomb cut-off	1.0 nm
Periodic boundary conditions	All direction (xyz)
Relative dielectric constant	infinity
Cut-off distance for short-range neighbor list	1.0 nm
Distance to start switching the Lennard-Jones Potential	0.8 nm
The cut-off distance for the Lennard-Jones Potential	1.0 nm

PME interpolation order	6
Dipole correction to the Ewald summation	off
Constraints	h-bonds
Neighbor searching type	grid
Soft-core alpha parameter	0.5
Power for lambda of soft-core function	1
Soft-core sigma for particles having C6 or C12 parameter smaller than soft-core sigma	0.3

6. FREE ENERGY CALCULATION

We utilize thermodynamics integration with the coupling factor method for free energy calculation.[17] We give a brief description of the methodology and procedures in our simulation, and various references, such as Ref.[[17]] provide detailed descriptions. In the coupling factor method, in order to calculate the free energy difference between two states, we manually change the Hamiltonian of a system, *H*, by adjusting the coupling factor λ using the soft-core strategy.[18] The free energy difference between two states, $\Delta G_{1\text{-}2}$, can be calculated as:

$$\Delta G_{1-2} = \int_{\lambda_2}^{\lambda_1} \langle \frac{\partial H(\lambda)}{\partial \lambda} \rangle \, d\lambda \quad \text{(S1)}$$

where the angle bracket denotes ensemble average. In the free energy calculation using thermodynamic integration, we calculate an ion dissolution process by “appearing” the ion in the solvent through gradually switching on the non-bonded interactions, including vdW forces and electrostatic interactions, between the ions and the solvent molecules (Figure S3). Appropriately formulating the λ-dependent non-bonded potential functions can conduct this process, with λ = 1 corresponding to the non-interactions state and λ = 0 corresponding to the fully interacting states. Around 30 discrete λ points are chosen between 1 and 0, and $\partial H(\lambda)/\partial\lambda$ is evaluated analytically in each MD simulation with different λ values. The detailed information of the λ-dependent potential functions can be found in Ref.[[17]].

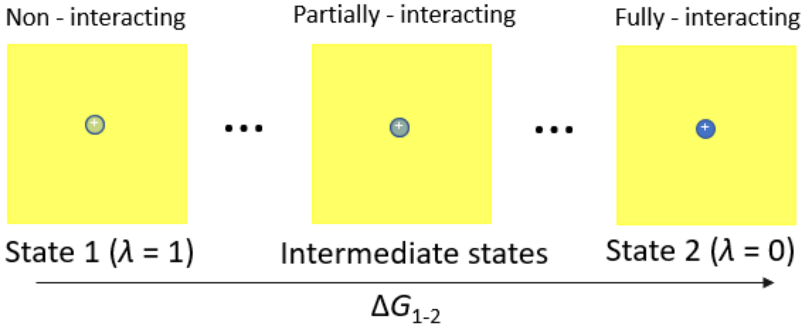

Figure S3. Schematics of “gradually appearing” a sodium ion in a solvent for calculating the free energy difference using MD simulations and thermodynamic integration. Here, λ is a coupling factor that tunes the interatomic interactions between the ion and solvent, with λ = 1 corresponding to the non-interactions state and λ = 0 corresponding to the fully interacting states.

In each simulation, the system is equilibrated with 20 ps constant volume (NVT ensemble), then a 100 ps further equilibration with constant pressure (NPT ensemble) is employed. Then, a 6 ns production run in the NPT ensemble is performed, where the derivative, $\partial H(\lambda)/\partial\lambda$, is evaluated and time-averaged. After collecting all the derivatives at each λ point, we use trapezoidal numerical integrations to calculate the free energy difference using Eq. (S1). The temperature is set 350K for all simulations.

We need to set a common reference to make a meaningful comparison between free energies. According to the definition, the solvation free energy of a solute molecule in a solvent is the value to describe the difference of the value between two states. One state is when it is in the crystalline solid, and the other is when it is dissolved in the solvent. In order to get the accurate value of the solvation free-energy value, we need to calculate two steps: breaking up the crystal lattice and dissolving the resulted gaseous ion. However, the breaking up of the crystal lattice step will be identical in all simulations, and the purpose in our simulation is to compare the tendency of the solute molecules being dissolved in different solvents (e.g., in water versus in directional solvents). Hence, only the free energy change in the second step is needed to calculate. All values from simulation are listed in Table S5.

Table S5. Free energy of solvation from simulation at 350K (77℃)

solute	solvent	free energy of solvation (kJ/mol)
TIP4P	TIP4P	-22.9
TIP4P	[emim][Tf_2N]	-26.5
[emim][Tf_2N]	TIP4P	-23.3*
[emim][Tf_2N]	[emim][Tf_2N]	-38.6*
NaCl	TIP4P	-699.5
NaCl	[emim][Tf_2N]	-677.7
TIP3P	TIP3P	-23.9
TIP3P	[emim][Tf_2N]	-24.0
NaCl	TIP3P	-709.6
[emim][Tf_2N]	TIP3P	-15.1*

* solvation free energy calculated involving [emim][Tf_2N] as a solute used ionic pair in vacuum (149.5 kJ/mol) as the reference state.

7. TERNARY PHASE SIMULATION

The procedure to set up the ternary phase simulation shown in Fig. 4 of the main text is as follows: (1) For saline water, 3.87% w/w of Na^+ and Cl^- are dispersed into the water phase. (2) For [emim][Tf_2N], the phase is performed in NPT ensemble. (3) NaCl solution and [emim][Tf_2N] are moved into the same box, and an equilibrium run is processed in the NPT ensemble to fully relax the structure at 350K. (6) A production run in NPT ensembles at 350K and 1 atm for 30 ns is carried out.

8. ENERGY FOR HEAT AND EXERGY ANALYSIS

We follow the procedure in Ref.[19] for the exergy calculation. The energy consumption is calculated based on the heat needed to increase the relevant mixture to desired temperatures. For a cycle between 20 °C (T_0) and a given top brine temperature (T_{TBT}), the thermal energy needed to heat the feed water-IL mixture to obtain a unit mass of produced fresh water is:

$$Q_{lab} = \left(\frac{C_{IL}}{Y_w} + C_w\right)(T_{TBT} - T_0) \quad (S2)$$

where Y_w is fresh water yield, and C_{IL} and C_W are respectively the specific heat of IL and water. We use the data from Ref.[20] to calculate C_{IL} of [emim][Tf_2N]. In larger scale process, heat recovery

scheme can be implemented via heat exchangers to harvest thermal energy from the hot stream to pre-heat the fee water-IL mixture. If we assume a practical heat exchanger efficiency (η_{HE}) of 0.8, which is common in industrial applications,[21] the net amount of thermal energy from the heat source to fuel the loop is:

$$Q_{ind} = Q_{lab}(1 - \eta_{HE}) \tag{S3}$$

To highlight the merit of using low temperature thermal energy, we calculate the exergy consumption, which depends on the top brine temperature of the process:

$$E = Q_{ind}\left(1 - \frac{T_0}{T_{TBT}}\right) \tag{S4}$$

Using Eqs. (S2) and (S3), Eq. (S4) can be re-written as

$$E = \left(\frac{C_{IL}}{Y_w} + C_w\right)(1 - \eta_{HE})(T_{TBT} - T_0)\left(1 - \frac{T_0}{T_{TBT}}\right) \tag{S5}$$

To estimate the energy needed for nanofiltration process, we refer to calculations in Ref.[22] and Ref.[23]. The minimum energy required to separate solute from water is related to solution temperature, water activity and water recovery rate, as shown in the following equation:

$$W_{min} = -RT \times \ln(a_w) \times \frac{\ln\left(\frac{1}{1-Y}\right)}{Y} \tag{S6}$$

where R, T, a_w is and Y respectively represent the gas constant, temperature, the water activity and the water recovery rate. By combining Eq. (S6) with the definition of osmotic pressure (Π),

$$\Pi = -RT \times \frac{\ln(a_w)}{V_w} \tag{S7}$$

where V_w stands for the molar volume of water, we obtain:

$$W_{min} = V_w \times \Pi \times \frac{\ln\left(\frac{1}{1-Y}\right)}{Y} \tag{S8}$$

If water recovery approaches zeros or if osmotic pressure is a constant value with water recovery, the theoretical minimum energy is:

$$W_0 = V_w \times \Pi \tag{S9}$$

If water recovery reaches 98.5% as the ratio shown in Ref[23], the theoretical minimum energy is:

$$W_0 = 4.27\, V_w \times \Pi \tag{S10}$$

9. PUMP ENERGY FOR HEAT EXCHANGER

The methodology is shown in Ref[24]. In order to maintain the running of the DSE desalination plant, three pumps are needed. The first pump is used for pumping the directional solvent from the low-temperature container to the high-temperature container, and the low-temperature directional solvent is heated in this process. The second is used for pushing the directional solvent from the high-temperature container to high-temperature container, and the high-temperature directional solvent is cooled in this process. The third pump is used for pumping the sea water into the system.

The exchanged heat in the heat exchanger can be expressed in the equation;

$$\dot{Q} = U \times A \times \mathrm{LMTD} \tag{S11}$$

In Equation (S11), $\dot{Q}$ is the heat transfer rate, U is the heat transfer coefficient which is

$$\frac{1}{U} = \frac{1}{h_{water}} + \frac{x_{thickness}}{k} + \frac{1}{h_{ds}} \tag{S12}$$

where h_{water} is the convective heat transfer coefficient for water, and h_{ds} is that for [emim][Tf_2N], and $x_{thickness}$ is the thickness of the wall of the pipe. By setting the operation temperatures to 20 °C and 45 °C in the DSE cycle, we can calculate the length of the pipe, L, which is then used for pressure drop calculation below.
Pressure drop, Δp, can be calculated as:

$$\Delta p = f \times \rho \times \frac{L}{D_h} \times \frac{v^2}{2} \tag{S13}$$

where f is the friction factor, ρ is the density of the flowing liquid, v is the velocity of the flow, L is the length of the flow in the heat exchanger, and D_h is the hydraulic diameter of the flow. Friction factor can be calculated from Churchill's equation[25]:

$$f = 8\left(\left(\frac{8}{\mathrm{Re}}\right)^{12} + \left(\frac{1}{(\Theta_1 + \Theta_2)^{1.5}}\right)\right) \tag{S14}$$

In equation S14, $\Theta_1 = \left[-2.457\ln\left(\left(\frac{7}{\mathrm{Re}}\right)^{0.9} + 0.27\frac{\varepsilon}{D}\right)\right]^{16}$ and $\Theta_2 = \left(\frac{37530}{\mathrm{Re}}\right)^{16}$, Re is the Reynolds number, and ε is the roughness of the surface. We can use the following equation to estimate the pumping power needed:

$$\dot{P}_p = \frac{\dot{V}\Delta p}{\eta_p \eta_e} \tag{S15}$$

where $\dot{V}$ is the flow rate of volume and η_p, η_e stand for the pump efficiency and the electrical motor efficiency, respectively.
In the calculation, we assume all the cylinder tubes are made of copper. We assume the inner diameter of the tube connected to pump 1 is 0.20m, and the diameter of the tube connected to pump 2 and pump 3 is 0.20m. The length of the tube connected to pump 1 is 15m, and the tube connected to pump 2, 3 have a length of 15m in total. Please refer to Ref. [[24]] for the indication of the pumps. The roughness of the tube is set to 0.34.[26] All calculations are based on a fresh water production rate of 1 kg/s, which needs a flow rate of IL of ~13.3 kg/s based on the water yield of the IL. With the pipe diameter of 0.20m, the IL flow velocity is 0.28 m/s. The density of [emim][Tf_2N] is 1520kg/m^3.[27] The thermal conductivity of [emim][Tf_2N] is taken from Ref[[28]]. Because we lack the convection heat transfer coefficient for [emim][Tf_2N], h_{ds}, we use the value of h_{water} as the value of h_{ds}, which we believe will not change the order of magnitude for calculation.

SUPPLEMENTARY REFERENCES

1. Weber, C. C., Masters, A. F. & Maschmeyer, T. Steric, hydrogen-bonding and structural heterogeneity effects on the nucleophilic substitution of N-(p-fluorophenyldiphenylmethyl)-4-picolinium chloride in ionic liquids. *Organic & biomolecular chemistry* **11**, 2534-2542 (2013).
2. Srour, H., Rouault, H., Santini, C. C. & Chauvin, Y. A silver and water free metathesis reaction: a route to ionic liquids. *Green Chem.* **15**, 1341-1347 (2013).
3. Kamio, E., Takenaka, A., Takahashi, T. & Matsuyama, H. Fundamental investigation of osmolality, thermo-responsive phase diagram, and water-drawing ability of ionic-liquid-based draw solution for forward osmosis membrane process. *J. Membr. Sci.* **570**, 93-102 (2019).
4. Tsuji, Y. & Ohno, H. Facile synthesis of thermally stable benzimidazolate-type ionic liquids. *Chem. Lett.* **42**, 527-529 (2013).
5. Rauber, D., Zhang, P., Huch, V., Kraus, T. & Hempelmann, R. Lamellar structures in fluorinated phosphonium ionic liquids: the roles of fluorination and chain length. *Physical Chemistry Chemical Physics* **19**, 27251-27258 (2017).
6. Tröger-Müller, S., Antonietti, M. & Liedel, C. Stability of the zwitterionic liquid butyl-methyl-imidazol-2-ylidene borane. *Physical Chemistry Chemical Physics* **20**, 11437-11443 (2018).
7. Fukumoto, K. & Ohno, H. Design and synthesis of hydrophobic and chiral anions from amino acids as precursor for functional ionic liquids. *Chemical Communications*, 3081-3083 (2006).
8. Frol̀ˆba, A. P., Kremer, H. & Leipertz, A. Density, refractive index, interfacial tension, and viscosity of ionic liquids [EMIM][EtSO4],[EMIM][NTf2],[EMIM][N (CN) 2], and [OMA][NTf2] in dependence on temperature at atmospheric pressure. *The Journal of Physical Chemistry B* **112**, 12420-12430 (2008).

9. Wang, H., Berton, P., Myerson, A. S. & Rogers, R. D. Double salt ionic liquids containing the trihexyl (tetradecyl) phosphonium cation: the ability to tune the solubility of aromatics, ethers, and lipophilic compounds. *ECS Transactions* **75**, 451 (2016).
10. Ahosseini, A. & Scurto, A. M. Viscosity of imidazolium-based ionic liquids at elevated pressures: cation and anion effects. *Int. J. Thermophys.* **29**, 1222-1243 (2008).
11. Berendsen, H.J. C., van der Spoel, D. & van Drunen, R. **GROMACS: A message-passing parallel molecular dynamics implementation**. *Comput. Phys. Commun* **91**, 43-56 (1995).
12. Jorgensen WL, Maxwell DS, Tirado-Rives J. Development and Testing of the OPLS All-Atom Force Field on Conformational Energetics and Properties of Organic Liquids. *J. Am. Chem. Soc.* **118**, 11225-11236 (1996).
13. Jorgensen, W., Chandrasekhar, J., Madura, J. **Comparison of simple potential functions for simulating liquid water**. *The Journal of Chemical Physics* **79**, 926 (1998).
14. Jorgensen, W. L., Chandrasekhar, J., Madura, J. D., Impey, R. W. & Klein, M. L. Comparison of simple potential functions for simulating liquid water. *J. Chem. Phys.* **79**, 926-935 (1983).
15. Darden, T., York, D. & Pedersen, L. **Particle mesh Ewald: An $N \cdot \log(N)$ method for Ewald sums in large systems**. *The Journal of Chemical Physics* **98**, 10089-10092 (1993).
16. Hess, B. P-LINCS: A Parallel Linear Constraint Solver for Molecular Simulation. *J.Chem.Theory Comput*, 116-122 (2008).
17. Straatsma, T.P. & McCammon, J. A. Computational Alchemy. *Annual Reivew of Physical Chemistry* **43**, 407 (1992).
18. http://manual.gromacs.org/documentation/.
19. Bajpayee, A., Luo, T., Muto, A. & Chen, G. Very low temperature membrane-free desalination by directional solvent extraction. *Energy & Environmental Science* **4**, 1672-1675 (2011).
20. Kabra, A., Becker, T. M., Wang, M., Ferreira, C. A. I. & Vlugt, T. J. Molecular Simulation of NH3/Ionic Liquid Mixtures for Absorption Heat Pump Cycles. (2017).
21. A.F. Mills. in *Heat Transfer* (Prentice Hall, New Jersey, 1998).
22. Elimelech, M. & Phillip, W. A. The future of seawater desalination: energy, technology, and the environment. *Science* **333**, 712-717 (2011).
23. Cai, Y. e. a. **Energy-efficient desalination by forward osmosis using responsive ionic liquid draw solutes**. *Environ. Sci.: Water Res. Technol.* **1**, 341-347 (2015).
24. Alotaibi, S., Ibrahim, O. M., Luo, S. & Luo, T. Modeling of a continuous water desalination process using directional solvent extraction. *Desalination* **420**, 114-124 (2017).
25. Churchill, S. W. & SW, C. Friction-factor equation spans all fluid-flow regimes. (1977).
26. Luke, A. Pool boiling heat transfer from horizontal tubes with different surface roughness. *Int. J. Refrig.* **20**, 561-574 (1997).
27. Krummen, M., Wasserscheid, P. & Gmehling, J. Measurement of activity coefficients at infinite dilution in ionic liquids using the dilutor technique. *Journal of Chemical & Engineering Data* **47**, 1411-1417 (2002).
28. Ge, R., Hardacre, C., Nancarrow, P. & Rooney, D. W. Thermal conductivities of ionic liquids over the temperature range from 293 K to 353 K. *Journal of Chemical & Engineering Data* **52**, 1819-1823 (2007).
